# Optimizing agricultural production for economic sustainability of sunflower across climatic zones

**DOI:** 10.1038/s41598-026-37479-x

**Published:** 2026-01-28

**Authors:** Hüdaverdi Gürkan, Hüseyin Bulut, Gerrit Hoogenboom

**Affiliations:** 1 Agricultural and Biological Engineering, University of Florida, Gainesville, FL USA; 2 Turkish State Meteorological Service, Ankara, 06120 Turkey; 3 University of Florida, Global Food Systems Institute, Gainesville, FL 32611 USA

**Keywords:** Decision support systems, Maximum profit, Planting date, Irrigation level, Fertilizer amount, Climate sciences, Ecology, Ecology, Environmental sciences, Plant sciences

## Abstract

Increasing global food demand and climate variability are placing unprecedented pressure on agricultural systems, necessitating a shift from generalized farming practices to site-specific precision management. However, a lack of long-term economic optimization studies for sunflower production in Türkiye limits the adoption of climate-resilient strategies. This study addresses this gap by utilizing the Decision Support System for Agrotechnology Transfer (DSSAT) to evaluate 1000 management scenarios across three distinct climatic regions (Edirne, Adana, and Konya) using 30 years of daily weather data (1991–2020). The analysis identified optimal planting dates, irrigation thresholds, and nitrogen rates to maximize economic profitability. Results indicated that optimal planting dates were March 20 for Edirne, April 30 for Adana, and May 10 for Konya. The economic optimum for irrigation start threshold was identified as 40% of Available Water Content (AWC) for Edirne and Adana, and 50% AWC for Konya, highlighting the value of managed deficit irrigation. Regarding fertilization, optimum profitability was achieved at 250 kg N/ha for Edirne and 300 kg N/ha for Adana and Konya. These optimized strategies significantly enhanced water productivity and ensured positive economic returns. The findings demonstrate the effectiveness of DSSAT in defining site-specific management protocols that reconcile economic viability with resource sustainability.

## Introduction

Currently, the main goals of agricultural production are high productivity, reduced production costs, environmental sustainability, and climate-smart agricultural practices. The increase in the world population has led to rapid degradation of natural resources and a reduction in cropland per capita, alongside an increasing annual demand for food^1^. This increase also poses a significant threat to food security.

The basic components of agricultural production are crop and cultivar genetic characteristics, soil physical and chemical conditions, management practices, and weather factors^2–5^. Most of these factors can be controlled by a producer. However, weather conditions are among the primary sources of uncertainty in open-field agricultural conditions. For this reason, climate is the most important unknown and main determining factor in crop growth and development for agricultural production^6^.

The interest and concern about the impacts of climate variability and climate change have increased in recent years. The concentrations of three key greenhouse gases in the atmosphere, carbon dioxide, methane, and nitrous oxide, reached record-high levels in 2024, the most recent year for which global consolidated data is available. According to the latest reports from the World Meteorological Organization (WMO), atmospheric CO_2_ concentrations have increased from approximately 278 parts per million (ppm) in 1750 to 423.9 ppm in 2024, an increase of 53%^7^. The increase in CO_2_ concentration in the Earth’s changing atmosphere directly affects the physiological processes and growth rates of crops. Moreover, changing precipitation patterns and the increasing frequency and intensity of weather-related hazards, such as droughts, floods, and storms, are indirect effects of climate change, also impacting plant processes. Increasing climate uncertainties trigger risks to agricultural production^8^.

Türkiye is located in the eastern Mediterranean and has diverse climatic conditions. While a Mediterranean climate prevails along the Mediterranean coast and in western parts of the country, a semi-arid climate prevails in central and southeastern Anatolia. The Black Sea coast, on the other hand, has a rainy, temperate climate throughout the year. Türkiye produces many of the crops that are grown in Mediterranean countries. In the Food and Agriculture Organization (FAO) 2023 global gross production value ranking released in 2025, Türkiye is ranked seventh^9^. According to reports by the Republic of Türkiye’s Ministry of Agriculture and Forestry (MAF), Türkiye is among the top four producers in the world of 20 agricultural commodities^10^. Sunflower is one of the world’s most important oil crops. In 2023, Türkiye ranked fifth among countries in the FAO for gross production value of sunflower seeds^11^. However, domestic consumption of sunflower oil is high, and the FAO 2023 report confirms that Türkiye is the world’s largest importer of sunflower seed^11^. Sunflower, with its widespread resistance and adaptability to rainfed conditions, is grown in almost every region of Türkiye. Because the production is based on rainfed agriculture, sunflower is thus sensitive to changes in climate variability.

Strategies to achieve agricultural adaptability to arid and semi-arid climates must ensure more efficient water use, a scarce resource in Mediterranean areas. In arid and semi-arid regions with rainfed conditions, changes are necessary in agricultural planning. For instance, while a temperature increase provides the opportunity to plant earlier in some regions, changes in the rainy season may delay planting^12^. Changes in the rainfall regime may cause drought when the plant is most sensitive to water, making irrigation increasingly common. Furthermore, access to irrigation water can often be curtailed during severe droughts. This situation requires planning regarding the irrigation method, time, and optimum irrigation amount in water-deficit conditions.

Planting time and irrigation requirements, which are affected by changing climatic conditions, directly impact productivity^13,14^. Fertilization is another factor that increases productivity, which is directly related to a farmer’s overall financial budget^15^. Additionally, fertilization efficiency is limited by soil properties, and additional fertilization does not always result in a higher yield^16^. Therefore, the most appropriate fertilization amount should be determined according to the soil structure of the production area. At the same time, other management practices also contribute to the determination of optimum production costs.

Soils are vital for supporting productivity. Temperature, precipitation, and moisture regime changes affect soil functions such as biomass production, biodiversity habitats, and water and air cleaning. Soil systems are fundamental to sustainable food security^17^.

In recent years, uncertainties that have increased due to climatic variability have been the most important risks in decision-makers’ production planning. Technological applications are widely used to reduce uncertainties, increase productivity, and decrease agricultural production costs. Many studies have been conducted to increase productivity and enhance the agricultural system. The main goal of most studies is to predict the final yield or any other harvestable product (biomass, etc.). One of the tools that can be used for these applications are crop simulation models.

Cropping simulation models have made significant progress in the last 40 years^18^. These models include numerical equations that illustrate the basic structure and generation mechanisms of carbon, water, and nitrogen balance, which are then implemented by a software application to simulate crop growth and development, nutrient uptake, water use, and total yield, as well as other plant characteristics and outcomes^19^. Cropping system models have been applied in a variety of operations around the world^20^. Using crop models to investigate the possible effects of climate change establishes a clear link among models, agrometeorology, and societal issues^21^. Crop simulation models can be helpful in many diverse aspects, from strategic planning for agricultural production on the farm to improving fundamental knowledge at the research scale^6,22^.

Agricultural production involves many uncertainties, with the primary goal being to maximize profits while minimizing costs. Climate variability has a direct impact on key factors such as planting or sowing dates, irrigation levels, and harvest timings. Previous studies have been conducted on optimizing water management^23–25^ determining optimum planting date^26–28^ nitrogen management^29,30^ and assessment of climate change effects^31–35^ using crop simulation models. Many previous studies have focused on optimizing single factors without considering economic trade-offs, long-term climate variability, or specific regional contexts, such as multi-location sunflower production in Türkiye. As a result, there is a significant need for assessments that simultaneously evaluate both biophysical and economic outcomes.

This study aims to address these gaps by using cropping simulation systems to identify management practices that are both agronomically optimal and economically feasible. The objectives of the study were (1) to determine the most appropriate planting date, (2) fertilizer level, (3) irrigation scenario to obtain maximum profit using a simulation approach for sunflower across three different climatic locations that represent the main sunflower production regions of Türkiye. To the best of our knowledge, this is the first comprehensive multi-location optimization study for sunflower in Türkiye that integrates planting date, irrigation, and nitrogen management with economic profitability analysis across 30 years of climate variability.

## Materials and methods

### Experimental data

This study examined the sunflower variety Ekllor, which is a registered and widely grown variety in Türkiye^36^, studied this variety in their research conducted during the 2015–2016 growing season in Konya, Türkiye. The experimental data were used for calibration of the CSM-CROPGRO-Sunflower model and included in the DSSAT sunflower cultivar characteristics file^35^.

This variety is high in oil, has a strong plant structure, and has wide adaptability. The study site was located in the Konya province (37°48’N, 32°30’E, 1031 m a.s.l.) of Türkiye. The experimental design was a Randomized Complete Block (RCB) with three replications. The plant and row spacings were set at 70 cm and 25 cm, respectively.

### The decision support system for agrotechnology transfer (DSSAT)

The Decision Support System for Agrotechnology Transfer (DSSAT) is built on calculations that estimate crop progression associated with the input data that includes weather and soil conditions, cultivar characteristics, and crop management options, as well as modeling structures in the soil-plant-atmosphere system^37–39^. DSSAT has been in use for over 30 years by researchers worldwide. DSSAT provides strategy evaluation options, including planting date, soil type, irrigation, fertilizer application, choice of variety, and economic analysis. The model can also be used to identify potential problems that may arise under various stress conditions (water, fertilizer, pests, etc.). DSSAT is used for a variety of purposes, including on-farm and precision management, as well as local evaluations of the effects of climate variability and change.

The primary module of the Cropping System Model (CSM) consists of five components, including weather, management, soil-plant-atmosphere, soil, and plant sub-modules. In the current version of DSSAT, different models are used for different plant groups, including legumes, vegetables, fiber crops, oil crops, grasses, grains, root crops, sugar/energy crops, and fruit crops.

Crop models predict daily growth, progression, and yield based on soil-plant-atmosphere interactions. DSSAT also allows users to select various equations for different components, such as evapotranspiration, soil evaporation, photosynthesis, soil layer distribution, infiltration, soil organic matter, and hydrology, for more accurate simulation. Among the most important benefits of DSSAT to decision-makers are its time, human resources, and cost reduction aspects.

### Model calibration

The CSM-CROPGRO-Sunflower model of DSSAT4.8.5 was used for conducting simulations in this study^39^. The model requires calibration based on crop cultivar using experimental data. Calibration is one of the most important stages of model studies. The model was calibrated and evaluated with data collected in 2015 and 2016^35^. While the cultivar was calibrated for Konya, the same genetic coefficients were applied to Edirne and Adana following standard DSSAT protocols where cultivar parameters remain constant across environments^37^. We acknowledge that local validation would strengthen confidence in absolute yield predictions. However, our focus on relative comparisons among management scenarios within each site minimizes this concern.

The model’s evaluation revealed that it could predict yield satisfactorily, with Normalized Root Mean Square Error (NRMSE) 1.3% for the irrigated treatment and 17.7% for the rainfed treatment, a d-index of 0.98, and a modeling efficiency (EF) of 0.93 in terms of the complete model accuracy^35^.

### Study regions and general features

**Fig. 1:**
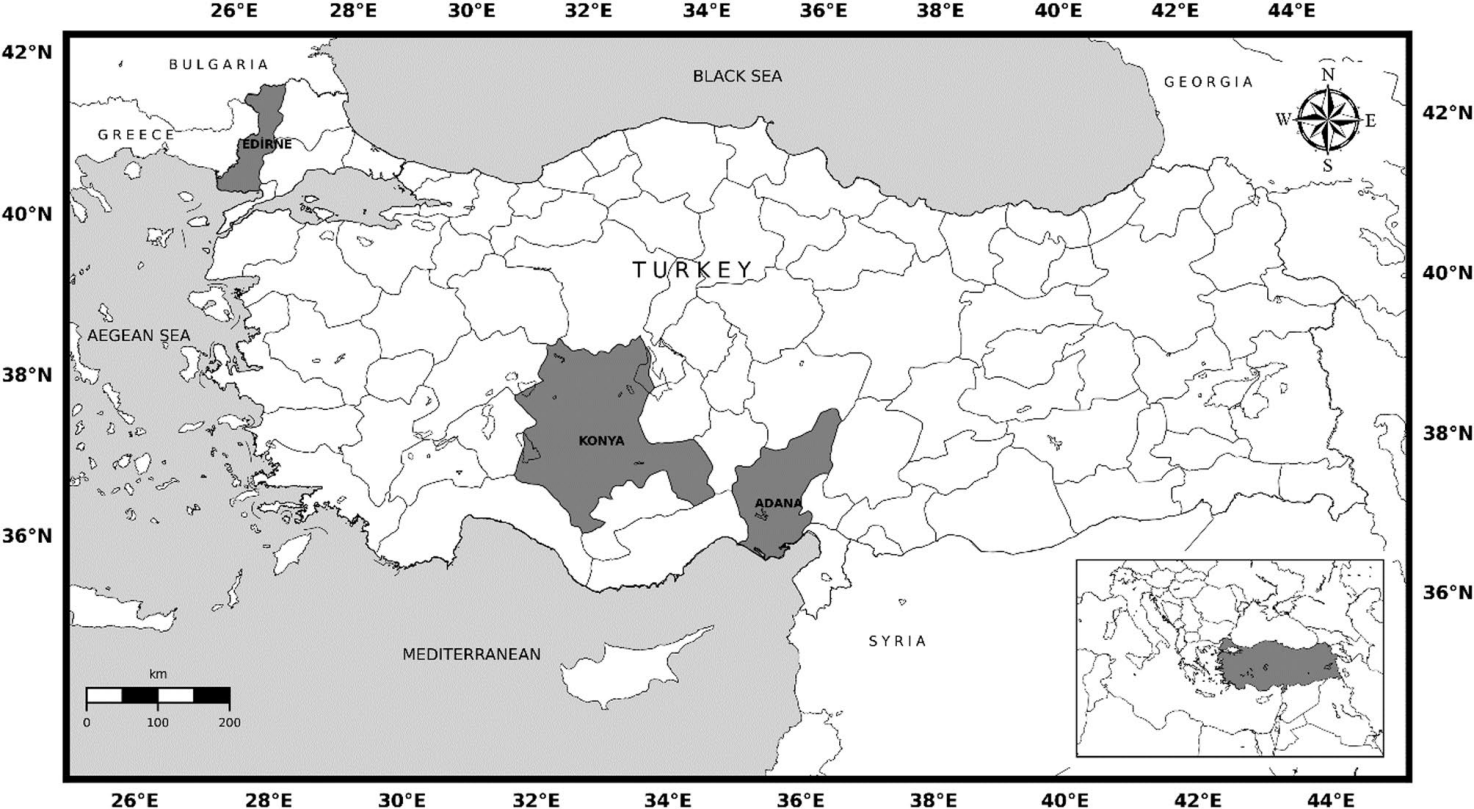
Study area, Türkiye.

Türkiye was selected as the site for this study. It has a wide variety of climatic characteristics due to its topographic structure and geographical location. There are limited irrigation opportunities in agricultural production, and the local weather variability makes the crop management decision process difficult for producers. Three locations (Konya, Edirne, and Adana) where sunflower production is carried out intensively and have different climate and soil characteristics, were selected in order to identify crop responses and different management practices (Fig. 1).

Edirne province is located in the Thrace region, which is the largest sunflower-growing region in Türkiye. Edirne is located in a region with a temperate climate and relatively more precipitation during the sunflower vegetation period. Traditionally, sunflower is planted throughout April. The second province, Adana, is located in the Cukurova region, where agricultural production takes place during all 12 months of the year. It has fertile soil and moderate climate conditions. According to the Köppen climate classification, a semi-arid climate prevails in the third region in the Konya province^40^. Konya has a continental and semi-arid climate. Traditionally, sunflower planting is done throughout May due to frost events in March and April.

One of the main inputs required for optimum modeling is a comprehensive soil profile. DSSAT contains several different soil profile databases for users. The soil profile database, which was derived from SOILGRID^41^, was used for the Adana province. The WISE (World Inventory of Soil Emission Potentials) soil profile dataset was used for the Edirne province^42^. The DSSAT soil database includes both of these soil profiles. The soil data for Konya province was obtained from an experimental field study^35^. The soil characteristics of the study areas in Edirne, Adana, and Konya, respectively, are clay loam, loam, and clay (Table 1).

**Table 1:** Soil physical and chemical characteristics.

Province	Depth (cm)	Clay (%)	Silt (%)	Sand (%)	LL (cm cm^− 1^)	DUL (cm cm^− 1^)	SSAT (cm cm^− 1^)	SBDM (g/cm^3^)	SLOC (%)	pH in water	SRGF
Konya	0–30	59.3	21.1	19.6	0.26	0.42	0.48	1.42	0.44	7.6	1.00
	30–60	61.7	21.1	17.2	0.27	0.44	0.50	1.47	0.30	7.9	0.75
	60–90	63.8	21.1	15.1	0.29	0.46	0.53	1.54	0.19	7.9	0.40
	90–120	64.0	21.0	15.0	0.29	0.45	0.52	1.46	0.12	7.9	0.30
	120–200	64.0	21.0	15.0	0.29	0.45	0.52	1.46	0.09	7.9	0.11
Adana	0–5	23.5	36.8	39.7	0.14	0.27	0.40	1.54	1.18	6.8	1.00
	5–15	25.5	36.0	38.5	0.15	0.28	0.41	1.56	0.99	6.9	1.00
	15–30	28.0	34.8	37.2	0.17	0.30	0.41	1.59	0.76	7.0	0.90
	30–60	30.5	33.5	36.0	0.18	0.31	0.42	1.64	0.49	7.1	0.75
	60–100	30.4	32.9	36.7	0.18	0.31	0.42	1.70	0.29	7.2	0.38
	100–200	28.8	32.6	38.6	0.17	0.30	0.41	1.75	0.16	7.4	0.11
Edirne	0–16	14.0	38.0	48.0	0.11	0.22	0.45	1.39	0.55	7.0	1.00
	16–34	31.0	27.0	42.0	0.17	0.29	0.43	1.48	0.24	6.9	0.61
	34–95	41.0	21.0	38.0	0.22	0.33	0.43	1.48	0.21	6.9	0.28
	95–120	38.0	11.0	51.0	0.21	0.31	0.38	1.59	0.07	6.7	0.12

LL= lower limit, DUL= drained upper limit, SSAT= saturation, SBDM= soil bulk density, SLOC= soil organic carbon, SRGF= soil root growth factor.

The initial soil conditions were established using representative soil profiles from each province. The baseline levels of mineral nitrogen for these soil series were set to create a low-residual-nitrogen environment. This approach allows for a more accurate evaluation of how crops respond to the specified nitrogen application rates.

### Climate dataset

**Fig. 2:**
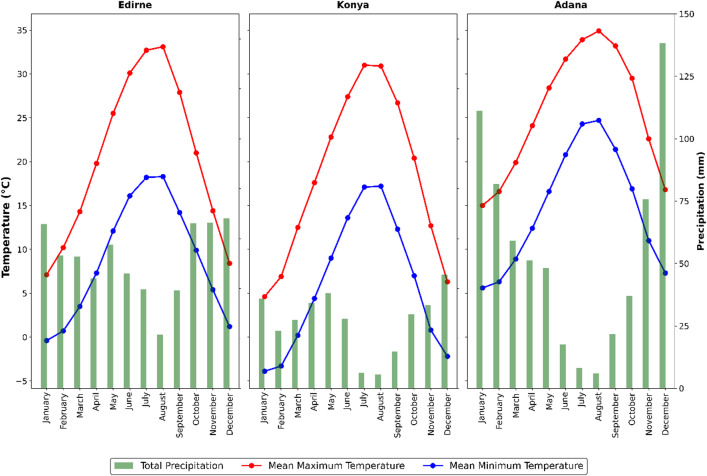
Climate analysis for the period 1991–2020 for each location.

The selected locations represent the country’s major sunflower production zones and distinct climatic gradients, which are necessary for national recommendations. Edirne and Konya are the leading provinces for sunflower cultivation, representing the Thrace and Central Anatolia regions, respectively, while Adana characterizes the warm-humid Mediterranean production zone. Konya is one of the most arid provinces of Türkiye, and Adana and Edirne provinces are in the Mediterranean climate zone. Thirty years (1991–2020) of daily weather data were used to reveal the agricultural production management analyses more accurately by considering climate variability. Also, the period 1991–2020 is the most recent climatological normal period. Daily observed weather parameters (minimum and maximum temperature, total precipitation, average relative humidity, total radiation, and average wind speed) were provided by the Turkish State Meteorological Service (Fig. 2)^43^.

### Management scenarios

In this study, a factorial design of management scenarios was created to determine the optimum production and maximum economic income by considering planting time, fertilization amount, and irrigation start threshold across three locations with distinct climatic conditions and soil structures.

Ten different planting dates were established at 10-day intervals, creating a 3-month planting window from March 1 to May 30. This wide temporal window was selected to cover the entire potential growing season, allowing the model to evaluate the trade-offs between early-season frost risks and late-season heat stress. Including early planting dates in continental regions like Konya, despite the known frost risk, was essential for quantifying the yield penalty associated with early planting and validating the safe threshold mathematically.

For the irrigation application, ten different scenarios were created, including a rainfed option and thresholds ranging from 10% to 90% of Available Water Content (AWC). In this study, 100% AWC represents field capacity. The automatic irrigation system was designed to activate when the soil’s available water fell below the specified threshold for each scenario (e.g., 40% AWC) and continued to operate until the profile reached 100% AWC (field capacity). Water balance calculations were based on the top 0–30 cm of the soil profile, and the drip irrigation method was chosen for all simulations to maximize application efficiency.

To assess the physiological and economic response of sunflower to nitrogen availability, ten fertilization scenarios were established. Application rates ranged from 30 kg N/ha to 300 kg N/ha, increasing in increments of 30 kg. Although standard agronomic recommendations rarely exceed 150–200 kg N/ha, the maximum rate of 300 kg N/ha was intentionally chosen to define the upper boundary of the yield response curve. This approach allows the study to explore the economic trade-offs under high-input conditions and explicitly identify the point where the marginal cost of fertilizer outweighs the marginal revenue from yield increases.

In the study, 1,000 scenarios were created to determine the most appropriate management application across all combinations of the three management variables (Table 2). Separate analyses were conducted for the three locations on an annual basis across 1,000 management scenarios and the 30-year analysis period from 1991 to 2020.

**Table 2:**
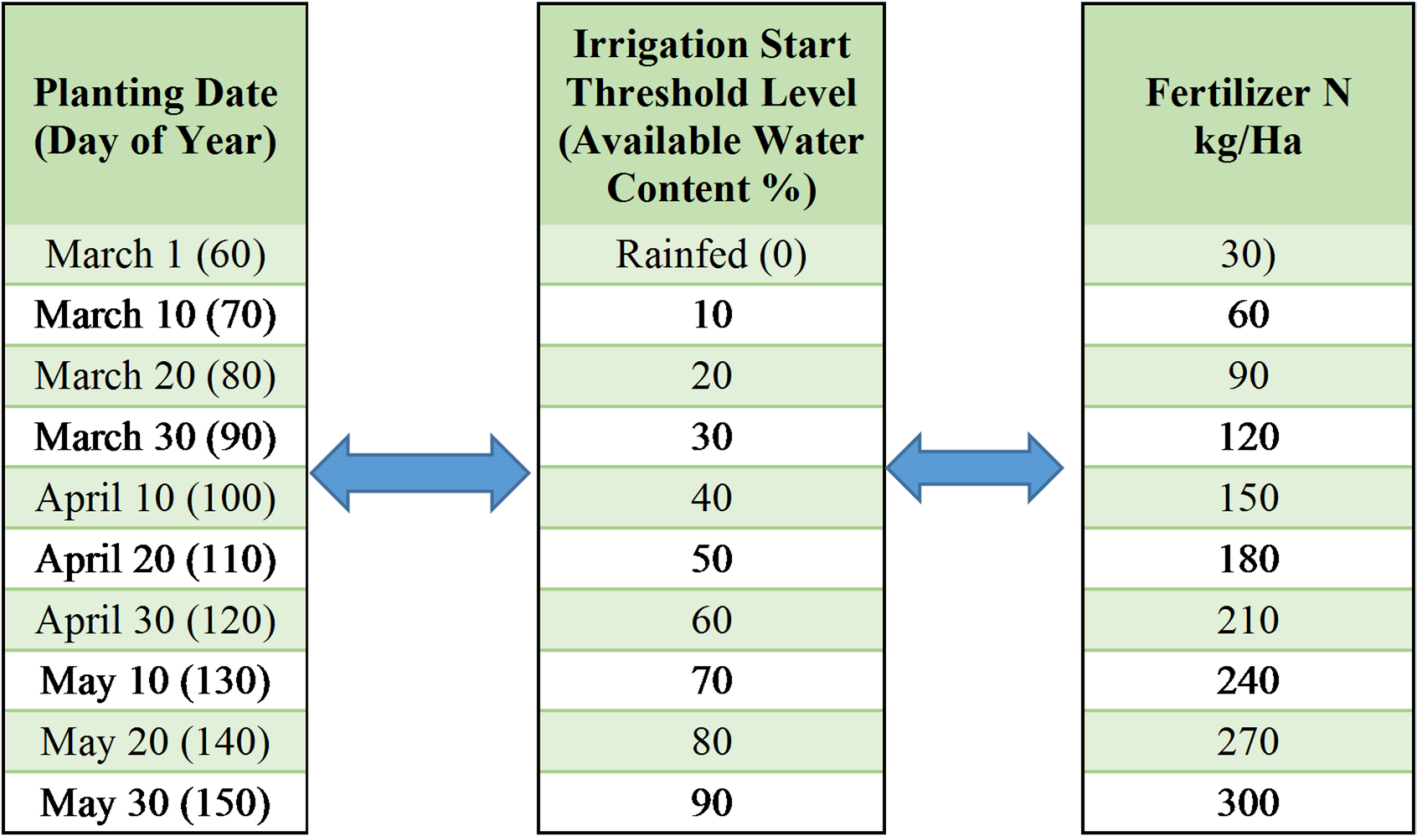
Sunflower management scenarios.

In the DSSAT economic analysis module, a profit-and-loss analysis can be generated at the end of production by defining the unit costs of the products and management applications used in the agricultural production process. In the model analysis module, costs for seed, planting, and irrigation, applications of N, P, and K fertilizers, organic amendments, harvesting, and grain prices can be defined in the system. In this study, all prices were expressed in US dollars (USD). The unit prices used in the economic analysis are given in Table 3. The economic analysis utilized the 2025 market prices published by the Edirne Commodity Exchange (ETB) and the Union of Chambers of Agriculture of Türkiye (TZOB)^44,45^.

An economic analysis was conducted using 2025 market prices to evaluate how well current management systems perform under historical climate variability. This approach follows the Agricultural Model Intercomparison and Improvement Project (AgMIP) standard protocols for assessing the sensitivity of agricultural systems to climate change^46^. By keeping economic parameters constant, we can isolate the biophysical risks associated with climate variability from external market fluctuations.

**Table 3:** Sunflower economic analysis components (data sources:^44,45^.

	Variable	Price (USD)
Expense	Base production cost (ha) (includes land preparation, sowing, and harvest operations)	50.0
	Seed (kg)	2.5
	Irrigation (mm)	0.15
	Cost per irrigation application ($/ha)	2.5
	N Fertilizer (kg)	1.25
	Cost per N fertilizer application ($/ha)	2.5
Revenue component	Harvested grain market price (tonne)	625.0

USD = US Dollar.

## Results

A total of 1000 scenarios were created to determine the optimal management scenario for maximizing profit based on planting date (PD), irrigation threshold (IST), and fertilization amount (FA), using 30 years of historical weather data.

### Optimum management for the Province of Konya

**Fig. 3:**
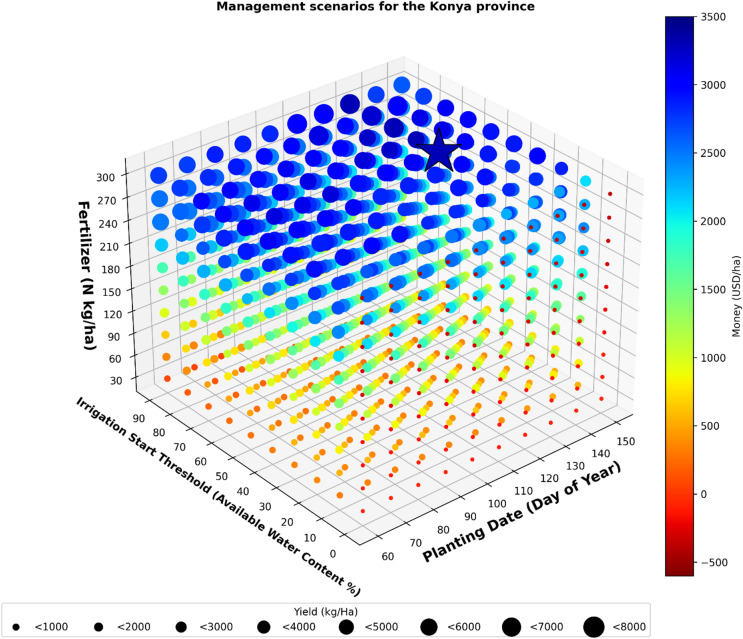
Management scenarios for the Konya province.

According to the analysis results for the province of Konya, the maximum profit was found for the scenario of 130 PD, 50 IST, and 300 FA (Fig. 3). It was determined that early May is the best time for sunflower planting.

The maximum profit obtained according to the selected scenario was on average 3300 USD, and with an average yield of 6730 kg/ha. In this scenario, which generated the highest average profit, the lowest profitability recorded was 1,543 USD, while the highest profitability reached 6262 USD over a thirty-year period.

The average analysis results of 1000 scenarios over 30 years showed that 102 scenarios are expected to result in economic losses. The maximum possible loss was 414 USD, and it would occur if the scenario of 90 PD, 0 IST, and 300 FA was applied.

### Optimum management for the Province of Edirne

**Fig. 4:**
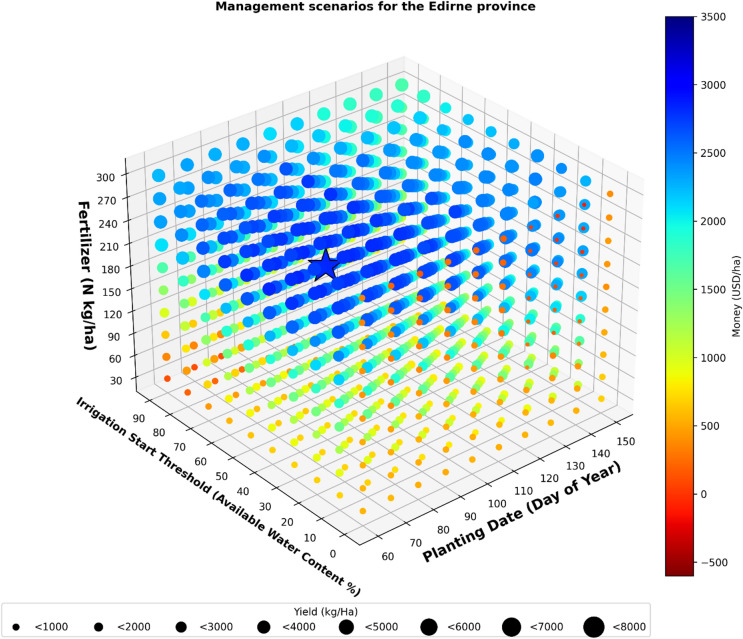
Management scenarios for the Edirne province.

The results of the analysis revealed that the optimum scenario for obtaining the maximum profit was 80 PD, 40 IST, and 240 FA (Fig. 4). The optimum planting date for maximum profit was during the second half of March, and not April, which is the current common planting date in the province of Edirne.

The analysis results showed that the maximum profit that might be obtained averaged 2839 USD for the province of Edirne. In this scenario, which generated the highest average profit, the lowest profitability recorded was 1349 USD, while the highest profitability reached 4420 USD over a thirty-year period.

In the scenario where the maximum profit was obtained, the average productivity was 5663 kg/ha. The maximum yield of 5787 kg/ha was 90 PD, 70 IST, and 300 FA. However, in the scenario where maximum yield was achieved, the profit amount was not determined as the scenario with the highest profit; instead, the profit was calculated to be 2650 USD.

The simulations indicated that out of 1000 scenarios analyzed over 30 years, only five scenarios would result in losses if implemented. The maximum potential loss was 111 USD, and it would occur if the scenario of 140 PD, 0 IST, and 300 FA were applied.

### Optimum management for the province of Adana

**Fig. 5:**
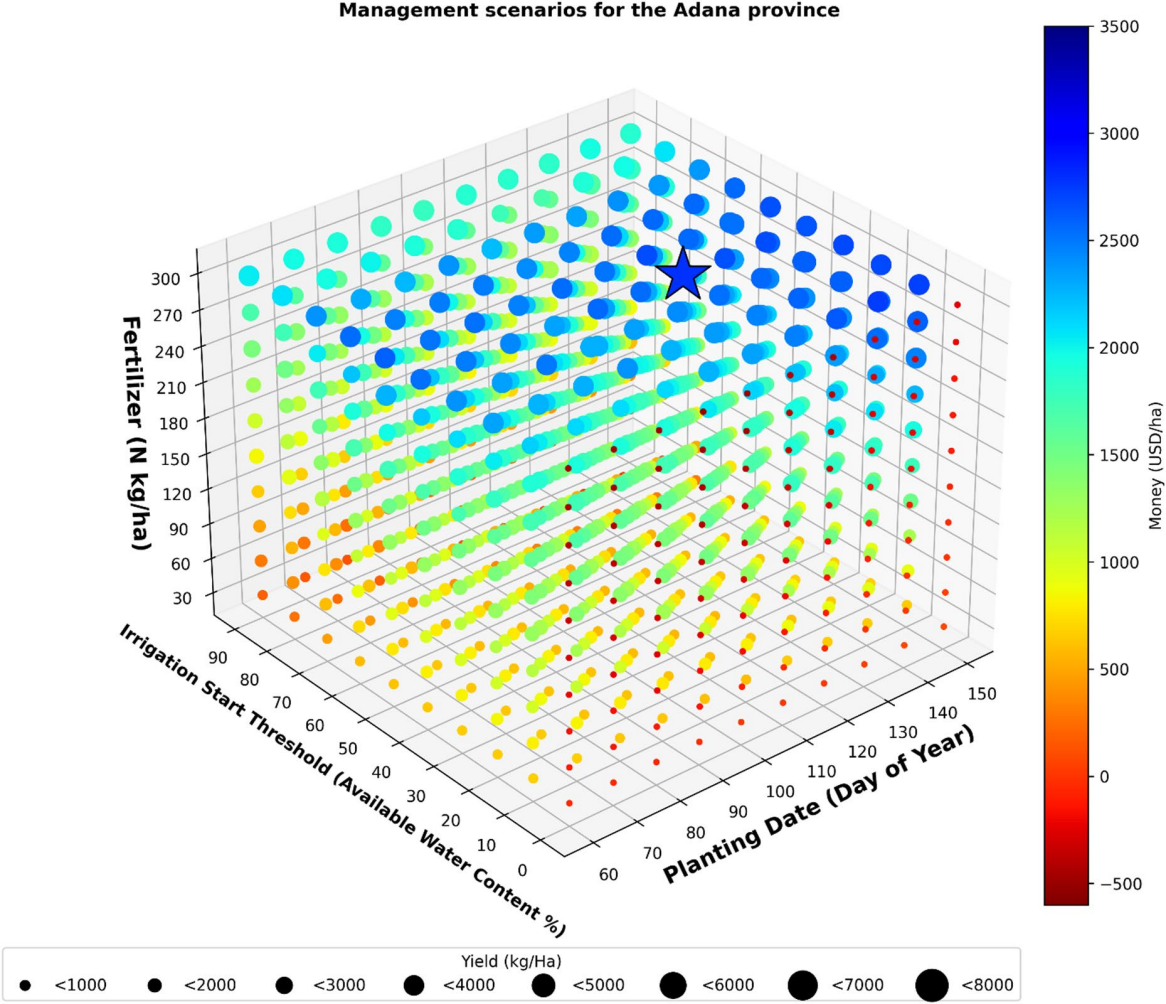
Management scenarios for the Adana province.

According to the analysis results for the province of Adana, the optimal scenario for maximizing profit was 120 PD, 40 IST, and 300 FA (Fig. 5). The optimal planting date for maximum profit was the end of April.

The analysis showed that the maximum profit averaged 2786 USD. In this scenario, which generated the highest average profit, the lowest profitability recorded was 980 USD, while the highest profitability reached 4928 USD over a thirty-year period.

In the scenario where the maximum profit was obtained, the average yield was 5725 kg/ha. The maximum yield of 5788 kg/ha was obtained from the scenario of 140 PD, 10 IST, and 300 FA. However, in the scenario where maximum yield was achieved, the profit amount was not determined as the scenario with the highest profit; instead, the profit was calculated to be 2736 USD. The simulations conducted over a span of 30 years analyzed 1000 distinct scenarios. The findings indicated that only 96 of these scenarios would result in a loss if executed. The maximum loss was 493 USD for the 90 PD, 0 IST, and 300 FA scenario.

The analysis shows that to maximize economic returns, it is essential to customize management practices to fit local environmental conditions instead of using a one-size-fits-all approach. Table 4 provides a comprehensive overview of the specific combinations of management practices, such as planting dates, irrigation threshold, and nitrogen usage, that led to the highest economic margins for each province.

**Table 4:** Summary of optimized management practices and economic outcomes.

Province	Planting date	Irrigation threshold	*N* rate (kg/ha)	Max. profit (USD/ha)
Edirne	March 20	40%	240	2839
Adana	April 30	40%	300	2786
Konya	May 10	50%	300	3330

### Irrigation productivity

**Fig. 6:**
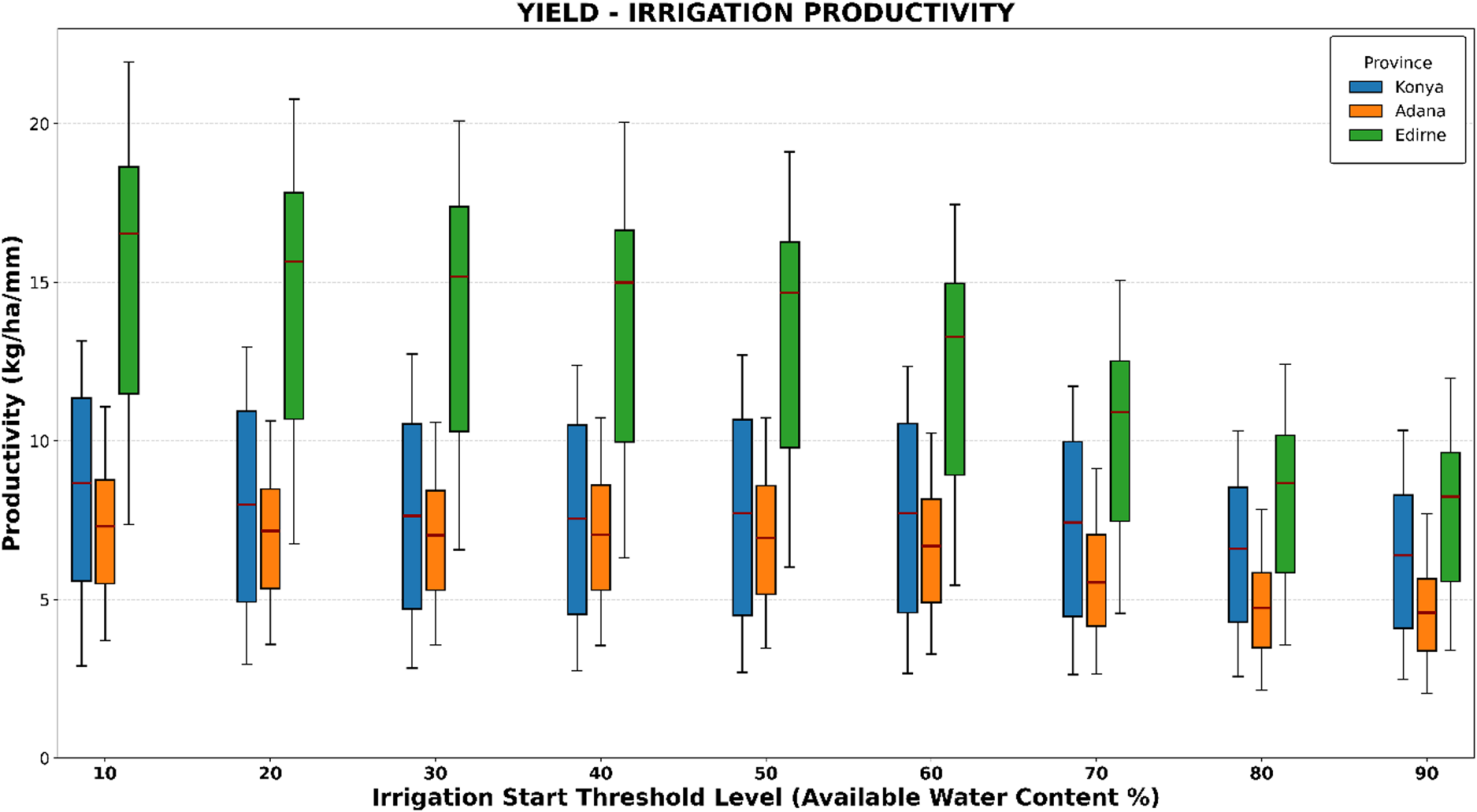
Yield- irrigation productivity (kg/ha –mm) analysis.

Productivity is defined as the ratio of output to input^47^. To effectively minimize agricultural production costs while maximizing income and gross margins, it is essential to evaluate the effects of various irrigation regimes on yield. For this analysis, yield-irrigation productivity (kg ha⁻¹ mm⁻¹) was assessed across three provinces at nine irrigation thresholds, which indicate the available water content at which irrigation should be initiated (Fig. 6).

The results showed that Edirne had the highest yield-irrigation productivity among all irrigation threshold levels. The peak yield-irrigation productivity was recorded at the 10% irrigation threshold across all three provinces: 15.4 kg ha⁻¹ mm⁻¹ in Edirne, 8.4 kg ha⁻¹ mm⁻¹ in Konya, and 7.1 kg ha⁻¹ mm⁻¹ in Adana.

For maximum profit, the optimal irrigation threshold was identified as 40% in Edirne and Adana provinces, while it was 50% in Konya province. At the 40% irrigation threshold, the average productivity was 13.9 kg ha⁻¹ mm⁻¹ in Edirne and 7.0 kg ha⁻¹ mm⁻¹ in Adana. In Konya province, the average productivity at the 40% irrigation threshold was 7.5 kg ha⁻¹ mm⁻¹.

### Fertilizer productivity

Fertilization is a key factor in enhancing agricultural productivity; however, it also represents a significant input cost. Therefore, determining optimal fertilization levels requires balancing biological efficiency with economic constraints. An analysis was conducted to evaluate fertilizer-yield productivity, specifically Nitrogen Use Efficiency (NUE), across three provinces to establish the optimal fertilization strategy.

**Fig. 7:**
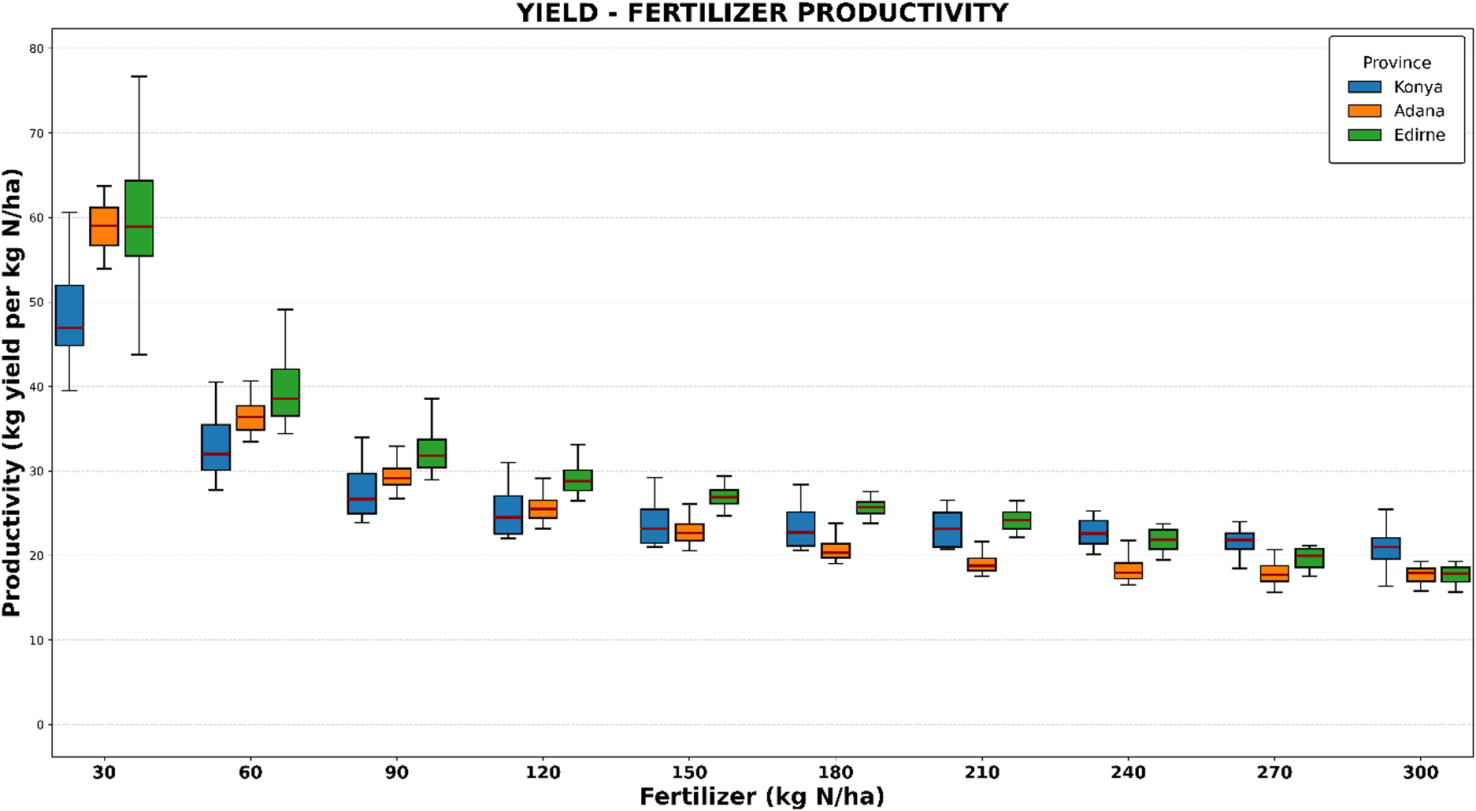
Yield- fertilizer productivity kg N/ha analysis.

The results revealed an inverse relationship between application rates and efficiency (Fig. 7). Consistent with the law of diminishing returns, all three locations reached peak NUE at the lowest application rate of 30 kg N/ha, exhibiting efficiency rates of 44.8 kg yield/kg N for Konya, 54.1 kg/kg N for Adana, and 58.7 kg/kg N for Edirne.

Critically, however, the fertilization rate that maximizes economic profit differs from the rate that maximizes agronomic efficiency. While low nitrogen rates yield the highest return *per unit* of fertilizer, they do not yield the highest total volume. In Edirne, the analysis indicated that applying 240 kg N/ha maximizes total profits (under the 80 PD, 40 IST, and 240 FA scenario), despite the NUE dropping to 20.4 kg/kg N. Similarly, both Adana and Konya achieve their highest net profitability at 300 kg N/ha. At this high-input level, the average NUE decreases to 16.2 kg/kg N in Adana and 19.0 kg/kg N in Konya (Fig. 7); yet, the substantial increase in total yield volume at these rates results in the maximum calculated economic return.

The simulation results highlighted distinct regional differences in nitrogen dynamics across application rates (30–300 kg N/ha). Nitrogen uptake increased linearly with application rates, ranging from 110 to 359 kg N/ha in Konya, 128 to 397 kg N/ha in Edirne, and 155 to 402 kg N/ha in Adana. The higher uptake in Adana is attributed to the region’s longer growing season and greater potential for biomass accumulation. Regarding N leaching, it was strongly influenced by the climatic zone. In the semi-arid conditions of Konya, leaching losses were negligible (~ 0 kg N/ha) throughout the simulation period, suggesting that moisture was the limiting factor and was fully utilized by the crop. Conversely, the Mediterranean climates of Edirne and Adana showed average leaching losses of 10 kg N/ha and 35 kg N/ha, respectively, driven by higher precipitation events and soil drainage characteristics.

## Discussion

The findings of this study, based on 1,000 simulated scenarios over a 30-year period (1991–2020) in three different climatic regions, indicate that closing the yield gap in Turkish sunflower production necessitates a transition from traditional practices to precision management tailored to specific climatic conditions. With a total of 90,000 the DSSAT cropping system model simulations, the results highlight the importance of adapting farming methods to overcome regional challenges.

The optimum planting dates were March 20 in Edirne, April 30 in Adana, and May 10 in Konya provinces, respectively. In contrast, the optimal planting date was earlier in the provinces of Edirne and Adana, where temperate climate conditions prevailed; in Konya, where continental climate conditions prevailed, the planting date differed. The determined optimum planting date matches Konya’s commonly preferred planting dates^48^. The optimum planting date determined in Adana, which has Mediterranean climate conditions, was close to the commonly preferred planting dates^49^.

The analysis identified distinct optimal planting windows based on regional thermal conditions: March 20 in Edirne, April 30 in Adana, and May 10 in Konya. These differences are explained by the need to balance soil temperature requirements for germination with the risk of frost. In the continental climate of Konya, the later optimal date of May 10 reflects the constraint of soil temperature; planting earlier carries a high risk of poor germination due to cold soils. In contrast, in Edirne, the analysis successfully captures the climatic shift driven by warming trends. While traditional practice suggests planting by late April to avoid spring frosts^50^, our analysis of data from 1991 to 2020 shows a significant decrease in frost frequency (Table 5).

**Table 5:** Edirne province frosty day analysis.

Edirne province	Total frost days		
Date/decade	1991–2000	2001–2010	2011–2020
March 20–31	25	1	1
April 1–10	4	11	1
April 11–20	0	1	0

Temperatures dropped below the − 4.0 °C biological damage threshold^51^, only once (-4.1 °C) in 30 years during the March 20 to April 20 window. This leads to the conclusion that early planting can maximize the vegetative period before summer heat sets in, resulting in a profit advantage of 200 USD/ha over the traditional April 20 date. This indicates that traditional frost risk guidelines in Edirne are becoming outdated due to climate change.

Due to the later planting date, the total amount of irrigation needed to reach the desired profit level was approximately 6.4% higher than when planting began on March 20. When March 20 was chosen as the planting date, the average total irrigation required was 299 mm. However, when the planting date was shifted to April 20, the average required irrigation increased to 318 mm. The results of the study indicated that selecting an appropriate planting date improves the efficient use of water resources, boosts crop yields, and enhances profitability.

The optimal irrigation starting threshold for achieving maximum economic profit was found to be 40% of available water content in Edirne and Adana, and 50% in Konya. These thresholds are lower than those reported in similar semi-arid environments. For example^52^, identified an ideal water content of 60–80% for maximum productivity in Spain. This difference highlights the distinction between agronomic and economic optimization. While maintaining soil moisture at 60–80% may maximize physical yields by reducing crop water stress, our economic analysis shows that the associated irrigation costs can lower net returns. Therefore, a managed deficit approach (40–50% threshold) provides the optimal balance between yield maintenance and input reduction for the cost structures present in the studied Turkish regions.

In Türkiye, rainfed farming conditions are common in sunflower production. The results of this study showed that supplemental irrigation can significantly increase productivity, as reported in previous studies^53^. The results of this study indicated that the maximum profit achievable under rainfed conditions was 362 USD for Edirne province. Based on the average results from a 30-year analysis of the Konya and Adana provinces, it was found that production under rainfed conditions would result in an average loss of 239 USD in Konya and 220 USD in Adana. In comparison, the maximum profit levels obtained under irrigated conditions were 3,330 USD for Konya, 2,839 USD for Edirne, and 2,786 USD for Adana. The results suggest that switching to an irrigated production model would be more profitable. The research results are also consistent with the results of irrigated field experiments, which showed an increase in sunflower productivity compared to the rainfed conditions^54–57^. The findings showed that the irrigated farming scenarios could bring in a 3.3, to 5.2 times higher income than rainfed farming. Sunflower yields tend to be low in rainfed conditions without the application of fertilizer. Based on a 30-year analysis, the highest possible profits recorded under rainfed conditions were 677 USD for Edirne, and 87 USD for Adana province. In Konya, rainfed conditions cause economic losses even under the best conditions.

Optimizing fertilizer application rates is crucial for enhancing agricultural profitability across diverse climatic regions. In this context, our research identified the most effective fertilizer rates to maximize profit across 10 fertilization applications (0–300 kg N/ha) tailored to three provinces in Türkiye. A comparative analysis of fertilizer yield productivity revealed distinct regional requirements. Edirne demonstrated the highest productivity levels, requiring a lower fertilizer rate to achieve maximum income compared to Konya and Adana. Specifically, while^58^, reported an average optimal rate of 197 kg N/ha for Edirne, our research suggests a higher optimum of 250 kg N/ha could further enhance crop yields.

In contrast, the optimal scenarios for Adana and Konya demanded significantly higher nitrogen inputs. For Adana, the maximum profitability was determined at 300 kg N/ha, resulting in a yield of 5,788 kg/ha. This exceeds the findings of^59^, who reported an optimal rate of 150 kg N/ha with a productivity of 3,360 kg/ha. Similarly, in Konya, the most favorable scenario yielded up to 6,730 kg/ha utilizing 300 kg N/ha and 540 mm of irrigation, representing a substantial increase over the field study by^36^, which achieved 4,646 kg/ha with significantly lower inputs.

While our economic analysis suggests that net returns could continue to rise at 300 kg N/ha in these specific high-yielding locations, we recommend capping fertilization at this level for the highest practical application rate. This threshold is selected to mitigate the well-documented inverse relationship between nitrogen supply and seed oil content. Previous research indicates that excessive nitrogen shifts plant metabolism toward protein synthesis at the expense of oil formation. For instance^60^, reported a significant decline in oil content from 46.2% to 40.6% as nitrogen rates increased to 240 kg N/ha. Similarly, studies conducted in Türkiye by^61^, observed that higher nitrogen applications reduced oil concentrations to as low as 36%, negatively affecting the industrial value of sunflower. Furthermore, excessive vegetative growth driven by high nitrogen rates can lead to lodging and delayed maturity, as noted by^62^. Therefore, 300 kg N/ha represents a prudent upper limit that balances the economic benefits of high gross yields with the need to maintain acceptable oil quality standards. Future studies should include a detailed analysis of oil content profiles at these super-optimal nitrogen rates (> 300 kg N/ha) to precisely quantify the economic trade-off between oil volume and oil quality.

Beyond quality concerns, the push for higher input presents broader sustainability challenges. In recent years, excessive fertilization and irrigation have often been favored to increase short-term profits. However, unconscious management practices can lead to diminishing returns and environmental degradation^63,64^. Excessive use of fertilizer and water jeopardizes system sustainability^65^, making effective planning an absolute necessity against food security risks. In this regard, the DSSAT model proves invaluable, facilitating agricultural production planning by allowing decision-makers to identify suitable scenarios without the time and cost associated with testing hundreds of options in field conditions^22^.

Furthermore, agricultural production remains one of the sectors most vulnerable to climate change^66^. Our findings indicate that the optimal planting date has shifted earlier due to changing climatic conditions. This aligns with the broader understanding that adequate planning for optimal planting times is essential for maximizing water use in arid and semi-arid zones, particularly as water access becomes increasingly challenging^67–70^.

Despite these climatic pressures, the optimal management scenarios demonstrated remarkable economic resilience. Throughout the 30-year simulation period, the best strategies for all three provinces consistently produced positive returns, resulting in a 0% probability of loss. Although climate variability inevitably affects annual profits, implementing optimized management practices significantly mitigates the risk of financial failure.

## Limitations

While this study offers a thorough economic optimization for sunflower management, there are several limitations to consider when interpreting the results.

First, concerning model validation, the DSSAT model was calibrated and validated using experimental field data from Konya. However, due to the lack of specific long-term experimental datasets for Edirne and Adana, the model assumed that the genetic coefficients of the cultivar remained constant across regions. Although this is a common modeling approach, lack of local validation for Edirne and Adana introduces uncertainty regarding the specific phenological responses to the unique microclimates of these provinces.

Second, the simulated yields represent ‘attainable yields’ rather than actual farm-gate averages. This distinction is critical when comparing results to regional statistics. Regional averages aggregate diverse management intensities, often dominated by rainfed production (particularly in Edirne and Adana), whereas this study simulates scenarios with optimized irrigation and nitrogen inputs. Furthermore, in this study, we assume conditions free from biotic stressors, meaning the reported yields do not account for losses due to weeds, pests, and diseases. Consequently, the discrepancy between these potential yields and the national average highlights a significant ‘Yield Gap’ caused by sub-optimal management and biotic stresses in farmer fields. However, the simulated values are consistent with high-input field experiments conducted in these regions, confirming their biological plausibility. For instance, in Konya^71^, achieved yields of 7310 kg/ha in field trials, closely mirroring our simulated maxima. Similarly, for the Thrace region^72^, reported experimental yields reaching 6060 kg/ha in Tekirdağ, confirming that the simulated 5663 kg/ha for Edirne is achievable under intensive management. Regarding phenology, while optimized planting dates for Konya and Adana aligned with traditional practices, the results for Edirne suggested a shift from the norm. This indicates that rising temperatures and extended frost-free periods in the Thrace region may offer new opportunities for earlier planting that traditional calendars have not yet adopted. Therefore, the yield and profit values reported here should be viewed as maximum potential targets achievable under optimal management”.

Third, soil properties introduce additional uncertainty. The simulations were based on representative soil profiles for each province; however, significant spatial variability exists within these regions regarding soil water content capacity and initial nitrogen content. Variations in these soil parameters could shift the optimum irrigation and fertilizer thresholds identified in the analysis.

Finally, the study relied on static economic parameters from 2025, applied to the historical climate record from 1991 to 2020. Although this method effectively isolates the impact of climate variability and management practices on profitability, it does not account for temporal market volatility, inflation, or year-to-year fluctuations in input costs. Additionally, the analysis was limited to a single standard cultivar under historical climate conditions. Future research should include partial-equilibrium economic models and future climate projections (e.g., CMIP6) to assess the combined risks of market volatility and long-term climate change.

## Conclusions

This study analyzed 1000 management scenarios over 30 years of climate data to highlight the importance of strategic planning for planting dates, irrigation thresholds, and fertilizer application to maximize crop profitability. The results showed that earlier planting dates, optimized irrigation strategies, and tailored fertilization can notably increase yields and profits, especially when transitioning from rainfed to irrigated systems. While these practices improve resource use, high nitrogen rates identified as economically optimal may require environmental trade-off analysis. Additionally, these strategies could help adapt to recent climate trends, though testing under future climate scenarios is necessary. The DSSAT cropping system model was essential for handling large data sets and efficiently testing multiple scenarios, offering data-driven recommendations for producers and decision-makers to ensure both economic viability and environmental sustainability. These findings are based on one cultivar (cv. Ekllor) and 30-year historical climate. On-farm validation, especially for Edirne and Adana, is recommended before large-scale adoption. Future work should evaluate recommendations under projected climate change scenarios.

## Data Availability

The data from this study are available from the corresponding author upon request. Some of the crop modelling files will also be incorporated into DSSAT and made available through the DSSAT GitHub repository in the near future.
